# Artificial intelligence in membranous nephropathy: transforming clinical management toward precision medicine

**DOI:** 10.3389/fmed.2026.1801351

**Published:** 2026-06-26

**Authors:** Lei Hua, Cuijie Zhao, Zhenhua Yuan, Hang Su, Mingyang Cai, Xianqing Ren

**Affiliations:** 1 Department of Pediatrics, The First Affiliated Hospital, Henan University of Chinese Medicine, Zhengzhou, Henan, China; 2 School of Pediatrics, Henan University of Chinese Medicine, Zhengzhou, Henan, China

**Keywords:** artificial intelligence, computational pathology, deep learning, membranous nephropathy, personalized therapy, precision medicine

## Abstract

Membranous Nephropathy (MN) is the leading cause of primary nephrotic syndrome in adults, characterized by significant clinical heterogeneity ranging from spontaneous remission to end-stage kidney disease. Current management of membranous nephropathy, which relies heavily on renal biopsy and static serological markers such as anti-PLA2R antibodies, often fails to predict individual disease trajectories. This limitation frequently leads to empirical immunosuppressive therapy with a trial-and-error approach. This review explores the transformative potential of Artificial Intelligence (AI) in reshaping MN management from evidence-based to data-driven and predictive medicine. We examine AI-driven innovations across the clinical spectrum: from computational pathology systems that automate glomerular morphometry with high objectivity and reproducibility, to non-invasive diagnostic models integrating radiomics and serology for non-invasive diagnosis. In therapeutics, we discuss machine learning algorithms that predict individual responses to Rituximab versus Cyclophosphamide, enabling personalized regimen selection. Furthermore, we highlight the role of AI in prognostic stratification, where dynamic in silico patient models and multi-omics integration unravel molecular subtypes and forecast renal survival with high granularity. While acknowledging critical challenges such as data silos, model interpretability gaps, and the need for global validation, we conclude that AI serves as a powerful augmentation tool. By synthesizing high-dimensional data into actionable insights, AI has the potential to facilitate increasingly predictive, preventative, and personalized care strategies in MN management.

## 1 Introduction

Membranous nephropathy (MN) represents the most prevalent cause of nephrotic syndrome in adults. The disease is characterized by subepithelial deposition of immune complexes on the glomerular basement membrane, resulting in progressive basement membrane thickening, podocyte dysfunction, and glomerular filtration barrier disruption (1, 2). The identification of circulating antibodies against specific podocyte antigens, most notably the M-type phospholipase A2 receptor (PLA2R) and thrombospondin type-1 domain-containing 7A (THSD7A), has fundamentally shifted the diagnostic paradigm from a purely histological to a serology-informed disease entity (3, 4). Despite these significant advances in understanding disease biology, clinical management remains largely empirical. The disease course is highly variable, ranging from spontaneous remission to persistent nephrotic-range proteinuria and progression to end-stage renal disease (ESRD) (5). Consequently, a uniform therapeutic approach is increasingly recognized as inadequate in the era of precision medicine. The ultimate goal is to move beyond this paradigm by tailoring immunosuppressive therapy—selecting the optimal agent, dose, and timing for each individual patient—through the deep integration of clinical, pathological, and molecular data.

Novel, multi-parameter algorithms mark a transformative breakthrough in MN diagnosis and management, poised to overcome current frameworks’ limitations. The 2021 Kidney Disease: Improving Global Outcomes (KDIGO) Clinical Practice Guidelines marked a significant advance by introducing a risk-stratified treatment algorithm (categorizing patients into Low, Moderate, High, and Very High risk) (6). Despite this progress, the algorithm’s foundation remains anchored in traditional clinical markers, primarily proteinuria and serum creatinine. These parameters are recognized as lagging indicators of underlying glomerular damage, often reflecting cumulative injury rather than providing a real-time assessment of disease activity. Several critical limitations stem from this reliance on conventional metrics. First, the observational approach and the consequent delay in immunosuppressive therapy risk irreversible nephron loss in patients with sub-nephrotic proteinuria (5). Second, while clinically useful, the existing risk strata lack the necessary granularity to predict an individual patient’s likely response to specific immunosuppressive agents, such as the choice between Rituximab and Cyclophosphamide (7). Third, the diagnostic gold standard of renal biopsy is inherently limited by inter-observer variability in interpretation and often cannot adequately capture the spatial heterogeneity of pathological lesions within the kidney (8, 9). Collectively, these shortcomings underscore a pressing unmet need for novel tools capable of enabling earlier, dynamic, and more personalized risk assessment that moves beyond the constraints of conventional clinical parameters.

**Figure 1:**
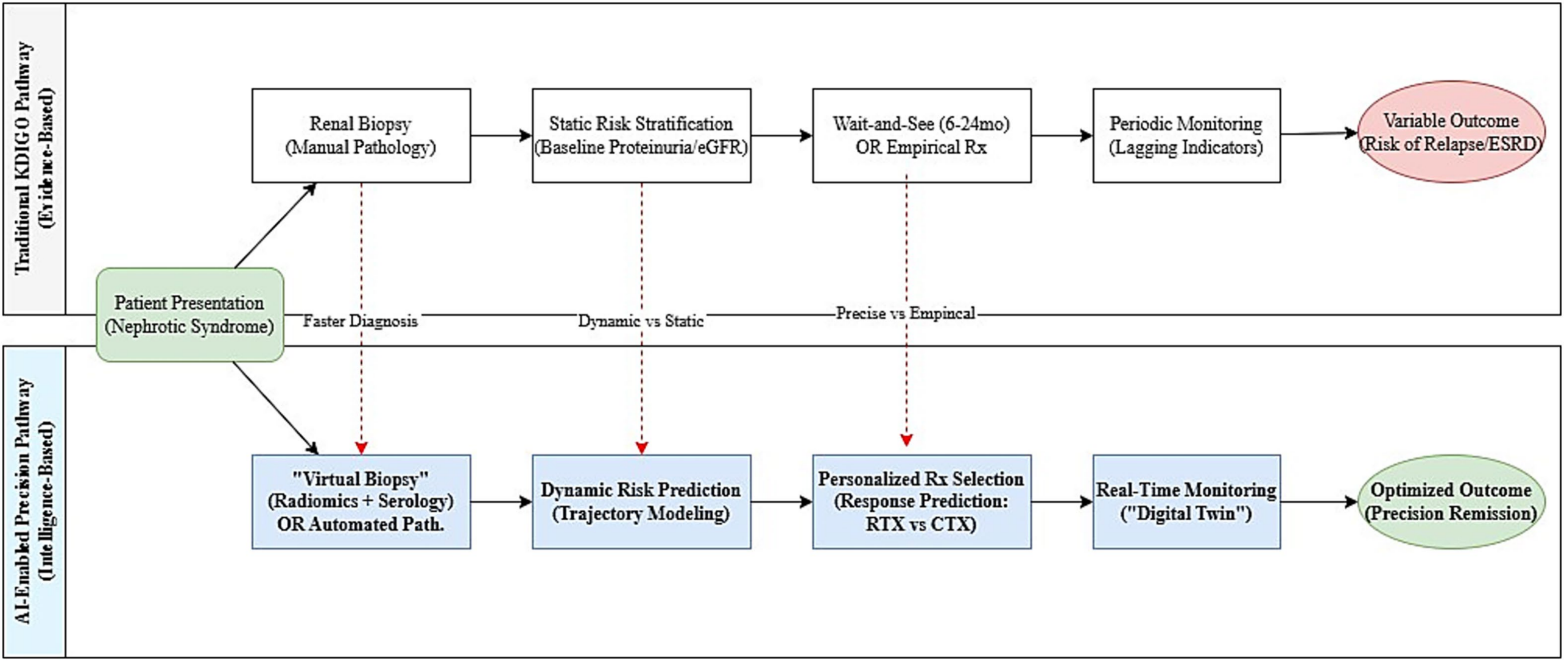
The paradigm shift: from evidence-based to intelligence-based management in membranous nephropathy.

Artificial Intelligence (AI), particularly machine learning (ML) and deep learning (DL), is rapidly transforming research and clinical practice in nephrology (10, 11). Unlike conventional statistical models that often rely on predefined assumptions, AI algorithms are adept at discerning complex, non-linear patterns within high-dimensional biological and clinical data. This capability is driving a fundamental evolution from a population-based, evidence-medicine paradigm toward a data-driven, predictive-medicine framework. This new framework, which we conceptualize as intelligence-based medicine, utilizes patient-specific data to generate individualized insights, moving beyond the averages derived from randomized controlled trials (Figure 1). Emerging applications—such as AI-powered computational pathology for automated glomerular analysis and integrated multi-omics for molecular subtyping—exemplify the potential of these tools to translate biological discoveries directly into clinical decision-support systems (9). Ultimately, by augmenting clinical expertise with computational intelligence, AI paves the way for a more proactive, precise, and personalized management strategy in MN. However, realizing this potential requires a critical appraisal of the current evidence, acknowledging that many AI models are still in their infancy and suffer from methodological limitations such as small sample sizes, risk of overfitting, and a lack of prospective clinical validation. To ensure a comprehensive and transparent overview, we conducted a literature search in PubMed for articles published up to February 2026. Search terms included combinations of “membranous nephropathy,” “artificial intelligence,” “machine learning,” “deep learning,” and “computational pathology.” Peer-reviewed original research, meta-analyses, and relevant review articles were prioritized for inclusion to synthesize the current state of AI applications in this field.

## 2 AI-driven diagnostic innovation: beyond the biopsy

The diagnosis of MN has traditionally relied on renal biopsy, an invasive procedure associated with inherent risks, including bleeding and infection (12). Furthermore, histopathological interpretation is limited by inter-observer variability and potential sampling error, which may not fully capture disease heterogeneity. Artificial intelligence is fundamentally reshaping this diagnostic paradigm. Through advancements in computational pathology, AI enhances the precision and reproducibility of tissue analysis (13). Concurrently, it enables the development of non-invasive diagnostic models by integrating radiomics, circulating biomarkers, and clinical data—an approach conceptually aligned with a “virtual biopsy” strategy.

### 2.1 Computational pathology and automated morphometrics

Computational pathology utilizes deep learning algorithms, particularly convolutional neural networks (CNNs), to analyze digitized whole-slide images (WSIs). This approach converts the subjective, qualitative assessment of renal histology into objective and quantifiable data, thereby enhancing diagnostic precision and reproducibility (14). For example, a 2025 study applied a hybrid CNN and Bidirectional Long Short-Term Memory (BiLSTM) model to analyze mouse kidney tissue Raman spectroscopy data, achieving 98% diagnostic accuracy for MN—outperforming other traditional machine-learning algorithms tested in the study. However, it is crucial to note that this model was not benchmarked against conventional evaluation by human renal pathologists, and it cannot yet be assumed that these preclinical findings will directly translate to human membranous nephropathy (15). These capabilities enable the automated identification and quantification of key histopathological features in MN, such as immune complex deposition and glomerular basement membrane alterations.

Beyond light microscopy, artificial intelligence has extended its analytical capabilities to transmission electron microscopy (TEM), enabling the automated, quantitative assessment of ultrastructural features essential for MN diagnosis. A representative 2025 study in JAMA Network Open detailed an AI system that automatically segments and quantifies glomerular basement membrane thickness, podocyte foot process effacement, and electron-dense deposits from TEM images (16). While this high-throughput morphometry offers a conceptual advance in mitigating inter-observer variability, the model was trained on a retrospective, single-center dataset. Without prospective clinical validation across diverse pathology laboratories, the consistency and generalizability of these automated metrics remain unproven. This high-throughput, automated morphometry provides objective and reproducible metrics that correlate with clinical disease severity and prognosis, thereby directly mitigating the challenge of inter-observer variability inherent in manual ultrastructural evaluation.

The application of deep learning is not limited to ultrastructural analysis. In another study focusing on glomerular pathological images from a small cohort of 45 patients, the VGG16 model achieved a detection accuracy of 71.9% at the glomerular level, while InceptionV3 attained a diagnostic accuracy of 73.3% at the patient level when differentiating MN from minimal change disease (MCD) and thin-basement membrane nephropathy (TBMN), However, without robust external validation, these findings primarily underscore the preliminary auxiliary diagnostic potential of such computational approaches for MN (8).

Further integration is being realized through multi-modal deep learning architectures. These models fuse complementary data from optical microscopy, immunofluorescence, and electron microscopy to achieve a more comprehensive diagnostic synthesis. For instance, a 2024 study demonstrated that such a fused model could accurately classify immune-mediated glomerular diseases, including MN, by learning and integrating discriminative features across different imaging scales and modalities (17).

### 2.2 The “virtual biopsy”: integrating serology and imaging

Although the identification of anti-PLA2R and anti-THSD7A antibodies has enabled a reduced reliance on renal biopsy in a subset of patients, a minority of individuals with MN are seronegative for known antigens, necessitating histological confirmation (18). Artificial intelligence shows promise in addressing this diagnostic gap by developing non-invasive predictive models that integrate multi-source data to estimate the likelihood of MN—an investigational approach conceptualized as a “virtual biopsy.”

A pivotal technology enabling this approach is radiomics, which involves the high-throughput extraction of quantitative features from standard medical images. Preliminary studies applying radiomic analysis to renal ultrasonography have demonstrated its potential to differentiate among glomerulopathies. For instance, a retrospective study involving a highly selected cohort of 68 patients employing LASSO regression and four machine learning classifiers on renal ultrasound radiomic features, finding that the Random Forest model best distinguished MN from IgA nephropathy with an area under the ROC curve of 0.764 in internal validation. It is important to acknowledge that an AUC of 0.764 represents only moderate discriminative ability. While this suggests that subtle, machine-discernible textural alterations in the renal cortex, might contain biological signals, this level of performance is currently insufficient to support a true “virtual biopsy” strategy or to replace histological evaluation (19).

To fully realize this diagnostic potential, radiomics is integrated into multimodal machine learning classifiers. Combined with key clinical variables, this fusion aims to enhance diagnostic utility, potentially offering a non-invasive tool capable of informing personalized therapeutic strategies. By combining radiomic signatures with key clinical parameters—such as proteinuria and estimated glomerular filtration rate (eGFR)—alongside serological profiles including anti-PLA2R and anti-THSD7A antibody status, researchers are developing robust predictive models (20). While such data fusion is theoretically sound, these models are predominantly derived from retrospective data. The lack of prospective, multi-center clinical trials means that their true clinical utility has not been established. These tools stratify glomerular pathology based on the likelihood of glomerular injury in patients with MN. In clinical practice, this stratification can guide decision-making: it may obviate the need for biopsy in patients with a high pre-test probability, such as those presenting with typical seropositive findings, while reliably identifying seronegative or clinically atypical patients for whom invasive histological evaluation remains essential (11). However, it is crucial to emphasize that “virtual biopsy” cannot yet fully replace histological evaluation, particularly for ruling out secondary etiologies (e.g., lupus nephritis) or superimposed pathologies that serology alone may miss. Furthermore, current multimodal models are frequently derived from single-center retrospective cohorts with limited sample sizes, raising significant concerns regarding selection bias and the risk of overfitting. Until these models undergo rigorous external validation across diverse populations, their generalizability and true clinical utility remain uncertain.

### 2.3 Precision phenotyping: distinguishing primary from secondary MN

Distinguishing primary (pMN) from secondary membranous nephropathy (sMN) is clinically essential, as sMN management targets the underlying etiology—such as malignancy, autoimmune disease, or infection—rather than relying primarily on immunosuppression. Conventional differential diagnosis often necessitates extensive and costly screening, posing a practical challenge in clinical settings (21).

Artificial intelligence demonstrates distinct value in identifying and elucidating the etiology of secondary MN, particularly in cases associated with malignancy. This capability is largely driven by machine learning (ML), which offers a powerful alternative by detecting subtle patterns within multidimensional data that may elude conventional assessment. For instance, recent bioinformatics-driven studies have employed ML to analyze transcriptomic data, seeking to identify distinctive gene expression signatures associated with malignancy-linked MN. A 2024 analysis highlighted RACGAP1 as a potential biomarker in lung adenocarcinoma-associated MN (22), while a 2025 study further delineated transcriptional markers specific to gastric cancer-related MN (23). Rather than serving as clinically validated tools for cancer detection, these bioinformatics analyses primarily identify candidate molecular pathways that may be shared between membranous nephropathy and selected malignancies. Prospective clinical validation is strictly required before such markers can be utilized to screen for occult neoplasms in clinical practice.

Beyond genomic approaches, ML models trained on routinely available clinical variables—such as age, gender, hematuria, and complement levels—have also shown high accuracy in differentiating pMN from sMN. These models can recognize complex, non-linear feature interactions—for example, the relationship between lower anti-PLA2R titers and malignancy risk in older adults—that are often missed by conventional clinical evaluation. Such AI-based clinical decision-support systems can integrate novel biomarker profiles—such as newly identified antigenic epitopes—to assess disease activity and prognosis. This facilitates risk-stratified management, helping to optimize the timing and intensity of therapy for individual patients (24).

## 3 Personalized therapeutic strategies

Current management of MN primarily involves immunosuppressive therapy for high-risk patients. The 2021 KDIGO guidelines recommend rituximab (RTX) or cyclophosphamide (CTX) as first-line agents. In practice, combination regimens are also frequently employed. However, treatment response is heterogeneous, with sustained remission rates ranging from 60 to 80% (25). This unpredictable efficacy, compounded by the narrow therapeutic index of many immunosuppressants, necessitates a “trial-and-error” strategy that carries risks of disease progression and treatment-related toxicity (26). AI has the potential to transform this field by facilitating predictive modeling for personalized therapeutic selection.

### 3.1 Predicting immunosuppressive response

Artificial intelligence is advancing the pretreatment prediction of therapeutic response in membranous nephropathy, primarily through the refined analysis of biological markers. While conventional metrics such as total anti-PLA2R antibody titers offer a baseline assessment, their ability to capture disease-specific immunological complexity is limited (27). AI models address this by integrating higher-dimensional data to improve prognostic accuracy. A 2024 study demonstrated that antibody profiles targeting specific PLA2R domains—including CysR, CTLD1, and CTLD7—predict clinical remission more effectively than total antibody levels alone (28). This was further extended by a 2025 study using a Random Forest algorithm, which showed that combining domain-specific antibodies such as PLA2R-CTLD678-IgG4 with epitope spreading data significantly enhances the prediction of 12-month proteinuria remission (29). Nevertheless, enthusiasm for these predictive algorithms must be tempered by their methodological constraints. Many of these studies rely on highly selected patient subsets and lack independent test cohorts, meaning the reported predictive accuracies may be inflated due to statistical overfitting and may not reliably translate to real-world clinical practice.

In parallel, AI is extracting predictive signals from histology. A 2025 study developed a classification model that couples hyperspectral imaging of pathological slides with a 1D convolutional neural network, identifying spectral features beyond conventional microscopy to predict Tacrolimus response (30). Similar deep learning approaches are being applied to predict resistance to rituximab, with the potential to guide early escalation to cyclophosphamide or next-generation anti-CD20 agents such as Obinutuzumab (31).

These efforts highlight ongoing explorations toward integrated clinical systems. For instance, the iRITUX trial protocol (2025) outlines an approach employing machine learning to predict rituximab underdosing risk and personalize dosing, aiming to improve remission rates over standard regimens (32). However, it is imperative to emphasize that this study is currently ongoing; no clinical results are yet available, and the efficacy of this AI-driven dosing strategy remains strictly investigational. Ultimately, by synthesizing multimodal data—from serological profiles to histopathological images—AI-driven stratification may extend beyond predicting single-agent response to elucidate broader mechanisms of therapeutic resistance in glomerular diseases, thereby informing precision treatment selection (33).

### 3.2 Real-time monitoring and dynamic regimen optimization

Membranous nephropathy exhibits dynamic disease activity that is poorly captured by intermittent conventional monitoring. The advent of continuous biosensing technologies—such as nanomaterial-based sensors for real-time creatinine measurement—now provides the requisite high-resolution temporal data (34). Leveraging these continuous data streams, the conceptual framework of AI-driven “Digital Twin” models has been proposed as a theoretical basis for real-time, integrated disease surveillance. It is crucial to emphasize that such models remain strictly hypothetical in the context of MN. In the future, if successfully developed and validated, these models could potentially integrate real-time biosensor data with longitudinal clinical inputs to dynamically track disease activity and personalize monitoring strategies, moving beyond episodic assessment toward adaptive, preemptive management (35).

Temporal deep learning architectures enable the construction of personalized disease trajectory simulations by continuously integrating longitudinal, multimodal data streams. These inputs include serial PLA2R antibody titers, trends in proteinuria, and patient-reported outcomes collected via digital platforms. As exemplified by studies utilizing Long Short-Term Memory (LSTM) recurrent neural networks, such models can dynamically forecast disease activity and progression (36). Critically, they demonstrate the potential to predict impending clinical relapses several weeks prior to the manifestation of overt signs, such as a significant rise in proteinuria. This early warning creates a vital therapeutic window for preemptive intervention, including the timely adjustment or initiation of maintenance immunosuppressive therapy.

While the direct clinical deployment of such systems in MN is still evolving, pioneering work in other chronic conditions provides a conceptual framework. For example, the SMART-CARE trial in heart failure showed that AI-based interpretation of wearable data significantly reduced hospitalizations (37). Translating this to nephrology, an algorithm-driven alert system for MN could integrate home-monitored parameters—such as daily weight, blood pressure, and patient-reported edema—to identify early deviations from baseline. This approach would enable proactive intervention, shifting MN management from reactive to preemptive care.

### 3.3 Balancing efficacy and toxicity: intelligent safety monitoring

In the traditional management of membranous nephropathy, immunosuppressive regimens have primarily relied on cyclic courses of corticosteroids combined with either cyclophosphamide or calcineurin inhibitors—agents associated with significant iatrogenic risks, including malignancy and infertility with cyclophosphamide (CTX) and substantial metabolic liabilities from prolonged steroid use (38). A key objective of precision medicine in this context is therefore to minimize such treatment-related harms.

Artificial intelligence models are capable of generating personalized risk profiles for adverse therapeutic events. By training machine learning algorithms on large-scale pharmacovigilance databases, it becomes possible to predict an individual patient’s susceptibility to specific toxicities, such as leukopenia and infection, based on pharmacogenomic markers, comorbid conditions, and concurrent medications (39). This approach is exemplified by recently validated models that utilize biomarkers like Torque Teno Virus levels to stratify infection risk in kidney transplant recipients (40)—While this evidence originates from the transplant setting, it provides a valuable methodological analogy for how similar data-driven safety monitoring could theoretically be explored in membranous nephropathy patients undergoing intensive immunosuppression. Consequently, a data-driven risk–benefit calculus can be established for each individual.

## 4 Prognostic intelligence and risk stratification

Accurate risk stratification forms the cornerstone of personalized management in membranous nephropathy. Conventional tools, such as the Toronto Risk Score, depend on static baseline parameters like proteinuria and eGFR, which may not adequately reflect the disease’s dynamic progression. In contrast, artificial intelligence enables a paradigm shift toward dynamic, high-dimensional prognostic modeling that continuously integrates evolving clinical data.

### 4.1 From static risk scores to dynamic trajectories

Static risk assessment models, while validated, have limited accuracy in predicting long-term outcomes for individual patients who may experience fluctuating disease activity. AI algorithms, particularly those based on joint modeling of longitudinal data and survival analysis, can dynamically update a patient’s risk profile as new data becomes available (41). For example, deep learning models can integrate time-series variables, including the decline rate of anti-PLA2R antibody titers or the velocity of proteinuria reduction, to estimate the probability of spontaneous remission versus disease progression. This approach is supported by a 2024 study in BMC Medical Informatics and Decision Making, which demonstrated that a machine learning–based dynamic online nomogram incorporating serial clinical parameters significantly outperformed static baseline models in predicting renal outcomes within a cohort of 232 patients. However, a critical limitation across this and similar studies is the frequent lack of direct, head-to-head comparisons with established clinical benchmarks, specifically the KDIGO risk stratification framework. Without demonstrating clear superiority or added clinical value over the current KDIGO criteria, the true utility of these AI models remains difficult to judge. Moreover, as the model was primarily evaluated using a specific regional cohort, broader external validation is necessary before widespread clinical adoption (42). Furthermore, a separate 2024 study developed a multivariable dynamic deep learning model that uses comprehensive longitudinal data to provide accurate and continuously updated prognostic assessments in IgA nephropathy (43), suggesting that similar dynamic approaches might be applicable to MN. Collectively, these dynamic trajectory–driven strategies represent an intriguing future direction that could hypothetically help clinicians to adjust monitoring frequency and treatment intensity in real time. However, shifting management from a rigid, schedule-based paradigm toward a responsive and individually tailored approach will require extensive prospective validation to prove clinical safety and efficacy.

### 4.2 Multi-omics integration: unraveling molecular subtypes

MN represents a spectrum of molecular entities rather than a single disease. To address this underlying heterogeneity, artificial intelligence—leveraging its cost-effectiveness in computational experimentation and capacity for multi-omics integration—offers a powerful analytical approach. The integration of genomics, proteomics, and metabolomics with detailed clinical phenotypes is thus essential to advance precise subtyping and personalized management in MN.

Artificial intelligence techniques, including unsupervised clustering and dimensionality reduction such as t-SNE and UMAP, can delineate distinct patient subgroups characterized by unique molecular signatures and prognostic trajectories. For instance, a pioneering study in a limited cohort of 37 patients applied desorption electrospray ionization mass spectrometry (DESI-MS) combined with machine learning to differentiate membranous nephropathy from other nephrotic syndromes (specifically IgA nephropathy and lupus nephritis) based on lipidomic profiles, While this identified molecular signatures correlating with disease activity, the model’s performance remains to be confirmed in independent external cohorts (44). By integrating multi-omics data, such AI-driven models are progressively revealing the underlying heterogeneity within MN and may help to identify high-risk phenotypes that could benefit from earlier and more targeted therapeutic intervention (45). It must be stressed, however, that these multi-omics clustering approaches are currently hypothesis-generating. The identified molecular subtypes lack prospective clinical validation to confirm whether tailoring interventions based on these AI-derived clusters actually improves patient outcomes compared to standard care.

### 4.3 Predicting kidney failure (ESRD) with high granularity

Accurately predicting the time to end-stage renal disease (ESRD) is essential for the timely planning of renal replacement therapy. Conventional Cox proportional hazards models are frequently constrained by their inability to capture complex, non-linear interactions among risk factors. In contrast, AI-driven survival models such as Random Survival Forests and DeepSurv have demonstrated superior performance in forecasting renal survival. These models can process high-dimensional interactions between key clinical variables like age and blood pressure, important pathological features such as the extent of tubulointerstitial fibrosis, and the individual’s response to initial therapy. Supporting this methodological advance, a 2024 study developed a dynamic survival prediction model that utilizes longitudinal clinicopathological data to forecast ESRD risk and validated its accuracy in a general chronic kidney disease (CKD) cohort. While not specific to MN, this approach serves as a proof-of-concept for how similar models could be adapted for MN patients (46). Similarly, an Australian cohort study employing a machine learning–based renal failure risk equation demonstrated its effectiveness in stratifying risk among patients with chronic kidney disease, thereby facilitating the development of tailored management plans (47). These models aim to generate personalized renal survival curves, potentially providing patients with more precise prognostic estimates and assisting clinicians in optimizing the timing of dialysis access placement or preemptive transplant evaluation (Table 1).

**Table 1:** Summary of key artificial intelligence models investigated in membranous nephropathy and related glomerular diseases.

Study (Year)	Application area	AI/ML algorithm	Cohort size & study design	Validation metrics (internal/external)	Key methodological limitations
Zhu et al. (15)	Diagnostic (Pathology)	CNN + BiLSTM (Raman spectroscopy)	*n* = 30 (Murine cBSA model); Preclinical	98% Accuracy (Internal only)	Preclinical animal model; no benchmarking against human pathologists; high risk of overfitting.
Basso et al. (8)	Diagnostic (Pathology)	VGG16, InceptionV3	*n* = 45 (Human biopsies); Retrospective	71.9–73.3% Accuracy (Internal only)	Small sample size; single-center; lacks external validation; limited generalizability.
Zhang et al. (19)	Diagnostic (Radiomics)	Random Forest, LASSO	*n* = 68 (MN vs. IgAN); Retrospective	AUC 0.764 (Internal only)	Small, highly selected cohort; moderate discriminative ability; lacks independent external validation.
Zhou et al. (28); Zhang et al. (29)	Therapeutic (Response Prediction)	Random Forest (Serology/Epitopes)	Variable; Retrospective single-center cohorts	Improved prediction of remission (Internal only)	Retrospective design; highly selected patient subsets; lacks prospective, geographically distinct external validation.
Wang et al. (42)	Prognostic (Risk Stratification)	Dynamic Nomogram (Clinical parameters)	*n* = 232; Retrospective	Outperformed static models (Internal regional validation)	Lacks direct head-to-head comparison with KDIGO criteria; requires broader external validation.
Mondal et al. (44)	Prognostic (Multi-omics)	DESI-MS + Machine Learning	*n* = 37 (NS patients); Retrospective	Identified molecular signatures (Internal only)	Very small sample size;hypothesis-generating only; lacks prospective clinical validation.
Christiadi et al. (46)	Prognostic (ESRD Prediction)	Random Survival Forests	General CKD cohort; Retrospective	Validated in CKD cohort	Not specific to MN; serves only as a methodological proof-of-concept for MN.

CNN, Convolutional Neural Network; BiLSTM, Bidirectional Long Short-Term Memory; LASSO, Least Absolute Shrinkage and Selection Operator; AUC, Area Under the Curve; IgAN, IgA Nephropathy; NS, Nephrotic Syndrome; CKD, Chronic Kidney Disease; ESRD, End-Stage Renal Disease.

## 5 Critical appraisal: challenges to clinical translation

While the potential of AI in MN is immense, the transition from “bench to bedside” faces substantial hurdles. Moving beyond proof-of-concept studies to routine clinical deployment requires addressing systemic challenges related to data infrastructure, algorithmic transparency, and validation rigor.

### 5.1 Data silos and heterogeneity in rare diseases

As a rare disease, MN suffers from fragmented data landscapes. High-quality datasets are often siloed within specialized tertiary centers, lacking the volume and diversity required to train robust deep learning models. Unlike common conditions such as diabetes where “Big Data” is readily available, MN datasets are “Small Data,” characterized by high dimensionality but low sample size.

Furthermore, Data heterogeneity presents a further significant technical barrier in MN research. Variations in renal biopsy staining protocols, differences across assay platforms for anti-PLA2R detection (e.g., ELISA versus CLIA), and inconsistent definitions of clinical remission across studies can introduce substantial bias. Models trained on data from a single institution often fail to generalize when exposed to the “noise” of real-world clinical practice (48). Addressing this challenge necessitates the establishment of large-scale, multicenter registries with harmonized data standards or the adoption of privacy-preserving federated learning approaches. Illustrating the latter, a 2024 study introduced “FedMoP,” a novel federated learning framework designed to handle non-IID (independently and identically distributed) data across multiple medical centers, enabling collaborative model training without compromising data privacy (49).

Complementing federated learning, transfer learning (TL) provides a promising strategy to address the limited data availability typical of rare diseases like MN. This approach involves pre-training deep learning models on large, well-characterized datasets from common related conditions—such as diabetic kidney disease or IgA nephropathy—to capture generalizable features of renal pathology, including glomerular detection and tubulointerstitial fibrosis scoring (50). The learned representations can then be fine-tuned on smaller MN-specific datasets, effectively transferring knowledge across domains. For instance, the 2026 “SegRenal” model demonstrated how an AI segmentation tool initially developed for frozen sections could be successfully adapted and validated across different scanning platforms and tissue preparations, highlighting the practical utility of transfer learning in bridging technical and clinical heterogeneity (9).

### 5.2 The interpretability gap: trusting the “black box” (XAI)

Deep learning models, particularly CNNs used in pathology, are often criticized as “black boxes” because their decision-making processes are opaque to human users. In nephrology, where therapeutic decisions involve potent immunosuppressants with serious side effects, “blind trust” in an algorithm is unacceptable (51).

Clinicians need to understand “why” a model predicts a high risk of relapse or resistance to Rituximab. The lack of interpretability remains a primary bottleneck for clinical adoption (52). Emerging Explainable AI (XAI) techniques, such as SHAP (SHapley Additive exPlanations) values and saliency maps, are beginning to bridge this gap (53). For instance, a 2025 multicenter study developed an interpretable machine learning model for predicting acute kidney injury following partial nephrectomy, explicitly using SHAP values to visualize the contribution of each clinical feature to the risk score (10). Although applied in a different clinical setting, this methodology illustrates how integrating such XAI tools into MN prediction models is essential to foster trust and human-AI collaboration (54).

### 5.3 External validation and generalizability

A major limitation of many AI models in nephrology is overfitting, wherein they perform well on internal training data but fail to generalize to external validation cohorts (55). This challenge is particularly acute in membranous nephropathy (MN), where ethnic variations in genetic susceptibility, such as “HLA-DQA1” alleles, and differing environmental exposures further contribute to biological heterogeneity (56). Consequently, a model trained primarily on a European Caucasian population may not accurately predict outcomes for Asian patients, who exhibit both a higher prevalence of MN and distinct disease trajectories (57). Therefore, rigorous external validation across diverse geographic and demographic populations is essential before any AI tool can be considered fit for clinical application (58). Supporting this imperative, a 2025 study in NPJ Digital Medicine underscored how demographic biases in training data can lead algorithms to systematically misrepresent outcomes in underrepresented racial and ethnic groups (59). Moving forward, research must prioritize multi-center, prospective validation trials to ensure that AI-driven solutions are equitable and generalizable across the global MN population.

## 6 Future horizons

The next decade will witness the evolution of AI from a research tool to an integral component of the nephrology ecosystem.

### 6.1 Multimodal fusion: the ultimate “digital nephrologist”

**Figure 2:**
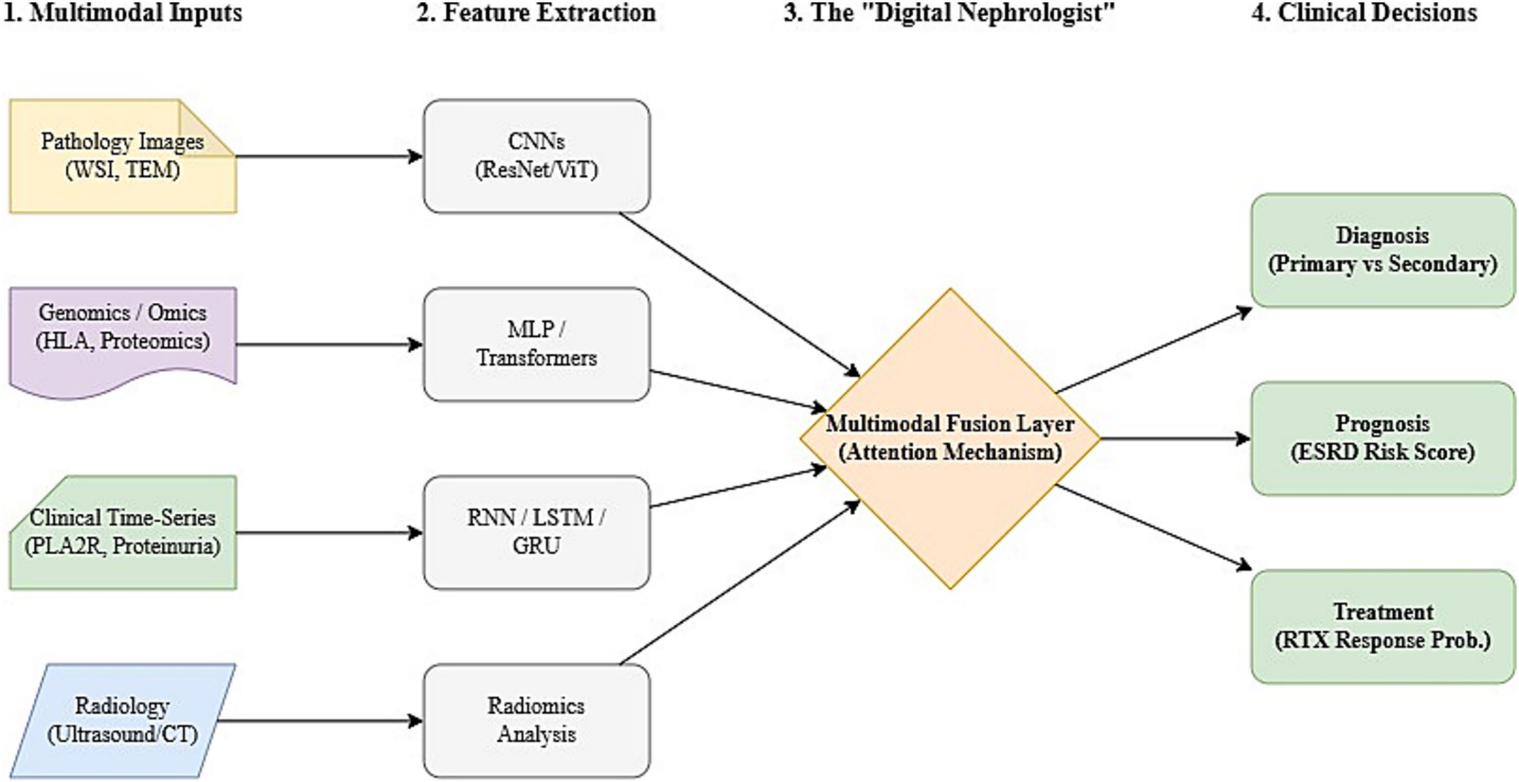
The “digital nephrologist”: a multimodal data fusion architecture.

Current artificial intelligence (AI) models in medicine predominantly focus on unimodal data analysis—such as histopathology, radiology, genomics, or clinical records alone. The next advancement lies in multimodal fusion, wherein integrated algorithms concurrently process diverse data types—including histopathological images, radiological scans, genomic profiles, and electronic health records—to construct a comprehensive patient profile (Figure 2). A 2025 study published in Scientific Reports illustrated the efficacy of this approach in renal cell carcinoma. The authors developed a fusion model that combined multiphase CT imaging with histopathological whole-slide images, which significantly surpassed single-modality models in predicting tumor recurrence (60). Similarly, recent frameworks have successfully merged electronic health records with imaging data to forecast chronic kidney disease (CKD) progression, underscoring the feasibility of multimodal integration in chronic disease management (61).

Applying this paradigm to membranous nephropathy (MN), a hypothetical “Digital Nephrologist” system could correlate glomerular ultrastructural features from transmission electron microscopy (TEM) with HLA genotypic data and serial anti-PLA2R antibody titers. Such a system could support the recommendation of individualized treatment strategies, dynamically adjusted over time. The synergy of “bits and biology” through multimodal AI promises to reveal novel dimensions in precision medicine, potentially exceeding the analytical limits of human cognition alone (62).

### 6.2 Federated learning for privacy-preserving collaboration

To overcome the data silo problem, Federated Learning (FL) is emerging as a game-changer. FL allows AI models to be trained across multiple institutions without sharing raw patient data. Instead of pooling sensitive records in a central server, the algorithm travels to the local data, learns from it, and sends back only the updated mathematical parameters (63).

This “privacy-preserving” architecture is particularly crucial for international collaborations on rare diseases like MN, where data governance regulations (e.g., GDPR, HIPAA) often hinder cross-border sharing. Comprehensive reviews have highlighted FL as a critical enabler for training robust medical AI models on diverse, decentralized datasets while maintaining strict patient confidentiality (64). By enabling a global network of hospitals to collaboratively train a “World MN Model,” FL can achieve the sample sizes necessary for high-performance deep learning (65).

### 6.3 Generative AI and large language models in nephrology

Beyond predictive modeling, Generative AI (GenAI) and Large Language Models (LLMs) like GPT-4 are poised to transform clinical workflows and patient engagement.

In the clinic, LLMs can automate the generation of discharge summaries and synthesize complex patient histories. A recent study in “*Nature Communications*” demonstrated that advanced LLM frameworks can provide meaningful assistance in 87% of evidence-based medical scenarios, suggesting a future where AI acts as a competent “second opinion” for diagnosis and management plans (66). For patients, GenAI can serve as a personalized education tool, translating complex medical jargon into plain language. However, recent evaluations caution that while LLMs provide high-quality medical information, the readability often exceeds the literacy level of the average patient (67). Future iterations must focus on “health literacy adaptation” to ensure that AI-driven patient education is truly accessible and empowering (68).

### 6.4 Bridging the regulatory chasm: from code to clinic

For AI algorithms to transition from research prototypes to clinical tools, they must navigate a complex regulatory landscape. Regulatory bodies like the FDA and EMA are evolving their frameworks (e.g., the EU AI Act) to address the unique challenges of “Software as a Medical Device” (SaMD), particularly for AI/ML-enabled devices that may change over time (69, 70). A 2025 analysis of FDA-authorized AI devices highlights the critical need for robust post-market surveillance to monitor performance drift (71). For MN, where disease definitions evolve, regulatory approval will increasingly rely on “Pre-determined Change Control Plans” (PCCP), which allow manufacturers to pre-specify future algorithmic modifications without triggering a new regulatory review for every update (70). Furthermore, ethical governance must ensure that AI tools do not perpetuate existing disparities in healthcare access. Transparent labeling of the model’s training population and limitations is essential to allow clinicians to judge its applicability to their specific patient (72). The path forward involves a “Total Product Lifecycle” approach, ensuring that AI tools remain safe, effective, and equitable throughout their clinical lifespan (73).

## 7 Conclusion

The integration of Artificial Intelligence into the clinical management of Membranous Nephropathy marks a pivotal transition from the era of “Evidence-Based Medicine” to the dawn of “Intelligence-Based Medicine.” By transcending the limitations of traditional biopsy and serology, AI is unlocking a new dimension of precision nephrology.

From automated computational pathology that quantifies glomerular injury with enhanced objectivity, to investigational “Virtual Biopsy” concepts aiming to non-invasively predict histological diagnosis, AI is beginning to influence diagnostic workflows. In the therapeutic arena, machine learning algorithms are replacing “trial-and-error” with personalized predictions of response to Rituximab and Cyclophosphamide, while “Digital Twin” technologies represent a future theoretical avenue for real-time, dynamic disease monitoring. Furthermore, the convergence of multi-omics data with advanced clustering techniques is unraveling the molecular heterogeneity of MN, identifying high-risk patient subgroups that demand early, aggressive intervention.

However, realizing this potential requires navigating significant challenges. We must overcome data silos through federated learning, demystify “black box” algorithms with Explainable AI (XAI), and rigorously validate models across diverse global populations to ensure equity.

Ultimately, AI is not a replacement for the nephrologist but a powerful augmentation tool. By synthesizing vast, high-dimensional datasets into actionable clinical insights, AI could eventually empower clinicians to deliver care that is increasingly predictive, preventative, personalized, and participatory. As we stand on this technological frontier, the future of MN management must focus on rigorously validating these tools to move closer to a reality where patients can receive more tailored and timely treatments.
